# Outcome of surgical treatment for metastatic bone disease of the distal femur: Observational single-center study of 47 patients

**DOI:** 10.1051/sicotj/2025062

**Published:** 2026-01-06

**Authors:** Adam Mellgren, Panagiotis Tsagkozis

**Affiliations:** 1 Södersjukhuset Sjukhusbacken 10 118 83 Stockholm Sweden; 2 Karolinska Institutet, Department of Molecular Medicine and Surgery 17177 Stockholm Sweden; 3 Department of Acute and Reparative Medicine, Karolinska University Hospital, Solna 17176 Stockholm Sweden

**Keywords:** Bone metastasis, Pathological fracture, Distal femur, Prosthesis, Osteosynthesis

## Abstract

*Introduction:* There is a paucity of data regarding the surgical treatment of distal femoral metastatic lesions. In this retrospective study, we aim to describe the outcome of surgery in this location and further analyze the findings based on the type of surgical reconstruction. *Methods:* 47 patients (48 fractures) who underwent surgery due to pathological fractures of the distal third of the femur, between 2000 and 2024, were included in the analysis. There were 29 prostheses and 19 osteosyntheses (10 plates, 9 nails). Local complications, implant revision rate, functional outcome regarding pain and ambulatory capacity, and overall survival were analyzed depending on the type of surgical treatment. *Results:* The complication pattern was different among implants used, with severe infections seen in prostheses (3/29 implants) and tumor recurrence in osteosynthesis (2/19 implants). In cases of osteosynthesis, failures resulting in revision surgery were documented only in cases of plate reconstruction (none when nails were used), resulting in a marginally higher revision rate (*p* = 0.14). Surgical treatment resulted in the restoration of the ambulatory capacity in 85% of patients, and pain levels were minor or moderate in 93%, without any significant difference between the surgical methods. Prostheses were used in patients with better overall survival (*p* = 0.015). *Discussion:* The patterns of local complications and their management differed between the different reconstruction techniques. Plate osteosynthesis had the highest risk for re-operation. The overall postoperative result was satisfactory, and functional outcomes were generally comparable. Patients with a good prognosis should be considered for reconstruction with a prosthesis when the bone quality does not allow nail osteosynthesis. Level of evidence: IV, retrospective study.

## Introduction

In the appendicular skeleton, the femur is the most common location of metastatic bone disease. Treatment of impending or completed pathological fractures of this major weight-bearing bone is generally surgical. Metastases in the distal part of the bone are far less frequent than in the proximal [1]. There are only two previous studies describing surgical treatment in this location, reporting on 29 [2] and 16 [3] patients respectively. Since the number of patients reported in the medical literature is low, there is a noticeable knowledge gap resulting in the absence of treatment recommendations [4]. In this location, a variety of implants can be used, and the associated complications, their severity, and management, as well as the functional outcome, are poorly described. This renders decision-making on the proper surgical strategy difficult.

Generally, reconstruction of metastatic skeletal defects relies on either osteosynthesis (OS) or endoprosthesis(EP) [5]. Each strategy has advantages and disadvantages, and there is no consensus among orthopedic surgeons regarding the most appropriate method to be used; treatment choices are individualized [6]. Further, OS can be performed with nails or plates, the latter often accompanied by cementation of bone defects. Nails tend to be used when the bone quality is good, allowing for good intramedullary stability and better cortical purchase of the comparatively lower number of screws available for their fixation [7, 8]. EP is generally reserved for patients with good condition and prognosis, and the use of survival estimation tools for patient selection is a sound approach [9–12]. Patients in good condition and with long survival have excellent functional outcomes when operated with an EP, with minor pain and very good ambulatory capacity [13, 14]. Since there is a definite improvement in the survival of patients with metastatic bone disease in the current era, an increasing number are considered for EP reconstruction [15].

This study aimed to analyze the surgical management of metastatic lesions of the distal femur, a location that is challenging due to the poor soft-tissue coverage and the extent of bone involvement often encountered. Bearing in mind the shortage of published data, we sought to provide evidence that can be used for informed decision-making and optimization of patient care. We primarily focused on the local complications depending on the type of implant used and how they were managed. Furthermore, we intended to report on the incidence of secondary surgery, including implant revision, and to describe the functional outcome.

## Materials and methods 

### Description of the cohort

Inclusion criteria were impending or completed fractures due to distal (below the isthmus) femoral metastasis treated surgically using an implant, between 2000 and 2024, with at least 6 months’ follow-up for living patients. Patients were identified in the institutional database, and further information was retrieved from the electronic files whenever possible. Two patients who underwent primary amputation, 3 who underwent curettage and cementation, one treated conservatively with a cast, and one with only 2 months’ follow-up were excluded. The epidemiological data are presented in Table 1.

**Table 1:** Baseline characteristics of a cohort of 47 patients/48 fractures surgically treated for metastatic disease of the distal femur.

Patient characteristics		Number
Gender	Female	24
Age at treatment	Median (range)	73 (52–88)
Primary diagnosis	Hematologic malignancy	10
	Renal cancer	10
	Lung cancer	6
	Breast cancer	5
	Prostate cancer	3
	Melanoma	3
Disease stage	Other	10
	
	Solitary bone metastasis	1
	Multiple bone metastases	13
	Generalized disease	33
Reconstruction	Plate osteosynthesis	10
	Intramedullary nail	9
	Endoprosthesis	29

### Surgery

Implants used were T2 or RFN retrograde nails (9 cases), lateral plates with a combination of locking and non-locking screws (8 cases), or both medial and lateral plates (2 cases). Cemented megaprostheses were 14 METS systems/Stanmore,14 Megasystem C/LINK, and one TC3 semiconstrained prosthesis (DePuy/Johnson & Johnson).

**Figure 1:**
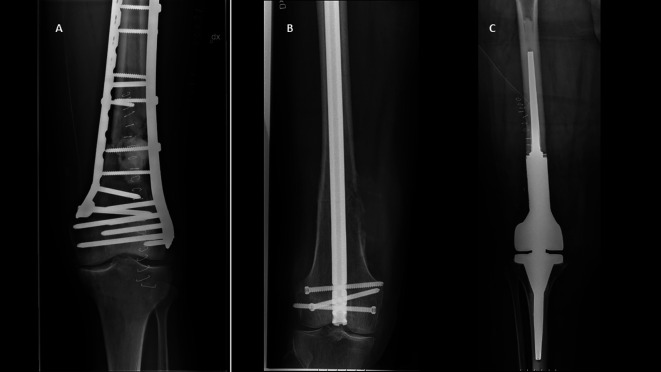
Representative cases of patients who underwent surgical treatment with distal femoral metastases. A. Plate osteosynthesis for lung cancer metastasis with displaced pathological fracture B. Retrograde nail osteosynthesis for colon cancer metastasis with non-displaced pathological fracture C. *En bloc* resection and megaprosthesis reconstruction for renal cancer metastasis with displaced pathological fracture.

In cases of nail OS, retrograde nails were inserted after a small medial arthrotomy, and locking screws were inserted percutaneously. Plates were placed through lateral and/or medial incisions of the quadriceps muscle. Cementation was performed in 7/10 cases of plate OS and in 1/9 cases of nail OS. In all EP cases, the tumor was resected marginally using a paramedial intraarticular approach, but after entering the lesion. All patients were mobilized without any restrictions. Representative cases are shown in Figure 1.

### Follow-up

The primary outcome was local postoperative complications. Secondary outcomes were: revision (defined as any type of surgery that involved removal or change of a part of the implant), any secondary surgical procedure, systemic complications, and functional outcome. The latter was assessed at an average of 6 weeks and in some patients 3–9 months postoperatively regarding the ambulatory capacity (subdivided in the following categories: no need of assistive device, use of crutches, use of walker or frame, confined to wheelchair or bedridden) and pain level (subdivided in the following categories: none, minor, moderate or severe).

### Statistics

Descriptive statistics are presented as absolute numbers and percentages, means and ranges, and medians with 95% confidence intervals. Comparison between categorical variables was done using Fisher’s exact test. Kaplan-Meier survival analysis was performed to evaluate the overall patient survival rate, with the log-rank test used for comparison between groups. Survival analysis with death as the competing event using the Fine and Gray method was used for implant revision rate estimation. All tests were double-sided. Statistical analyses were performed in SPSS (version 29, SPSS Inc., Chicago, IL) and SAS on Demand for Academics.

## Results

### Local post-operative complications and their management

Local complications and their management differed among the different surgical methods. In total, local complications were documented in 14 sites (29%). Both groups had a similar risk for infection (observed in 3 patients in each group). However, infections in cases of OS could be easily controlled with antibiotics (one patient with plate OS) or simple wound debridement and antibiotics (one plate OS and one nail OS). In EP patients, all 3 infections led to surgery with implant revision.

Tumor recurrence was reported in 2 patients in the OS group (both plate OS), leading to revision surgery (one conversion to a megaprosthesis and one amputation). There was one case of tumor recurrence in the EP group, which was treated with radiotherapy. Technical errors were noted in one EP patient (an implant part that was not inserted during the primary operation) and one plate OS (joint penetration of the screws). One patient in the OS group and one in the EP group had transient peroneal nerve palsy. One patient with an EP had a periprosthetic fracture.

### Revision surgery

**Figure 2:**
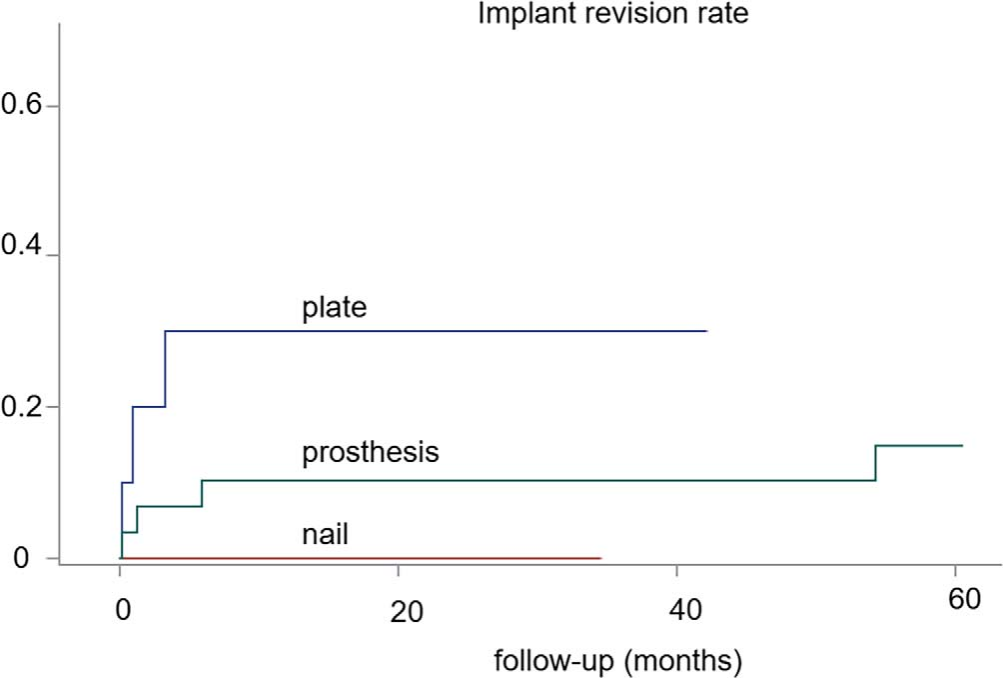
Revision rate for 47 patients/48 fractures surgically treated for metastatic lesions of the distal femur, depending on the type of implant used.

The surgical revision rate was marginally higher in cases of plate OS (*p* = 0.144). Revision surgery was performed in 8 cases. Three in OS (all in cases of plate reconstruction), 2 of them due to tumor progression and one due to technical error, as mentioned in the previous paragraph. In EP, 3 were due to infection, one due to a technical error, and one due to a periprosthetic fracture. The revision rate in a survival analysis with death as a competing risk is shown in Figure 2. Secondary amputations were performed in 2 patients: one patient treated with plate OS, where the tumor recurred, and one EP patient who suffered an infection that could not be controlled after revision of the implant.

### Systemic post-operative complications and oncological outcome

Patients with a good prognosis were more likely to undergo an EP. Median overall survival for all patients in the cohort was 8 months (95% confidence interval: 3–13 months). Patients treated with EP had a median survival of 17 (9–25) months compared to 3 (1–5) months for the group treated with OS (*p* = 0.015). The 30-day post-operative mortality was 11%. Systemic complications were documented in two patients in the OS group, and both were cardiovascular incidents.

### Functional outcome

**Figure 3:**
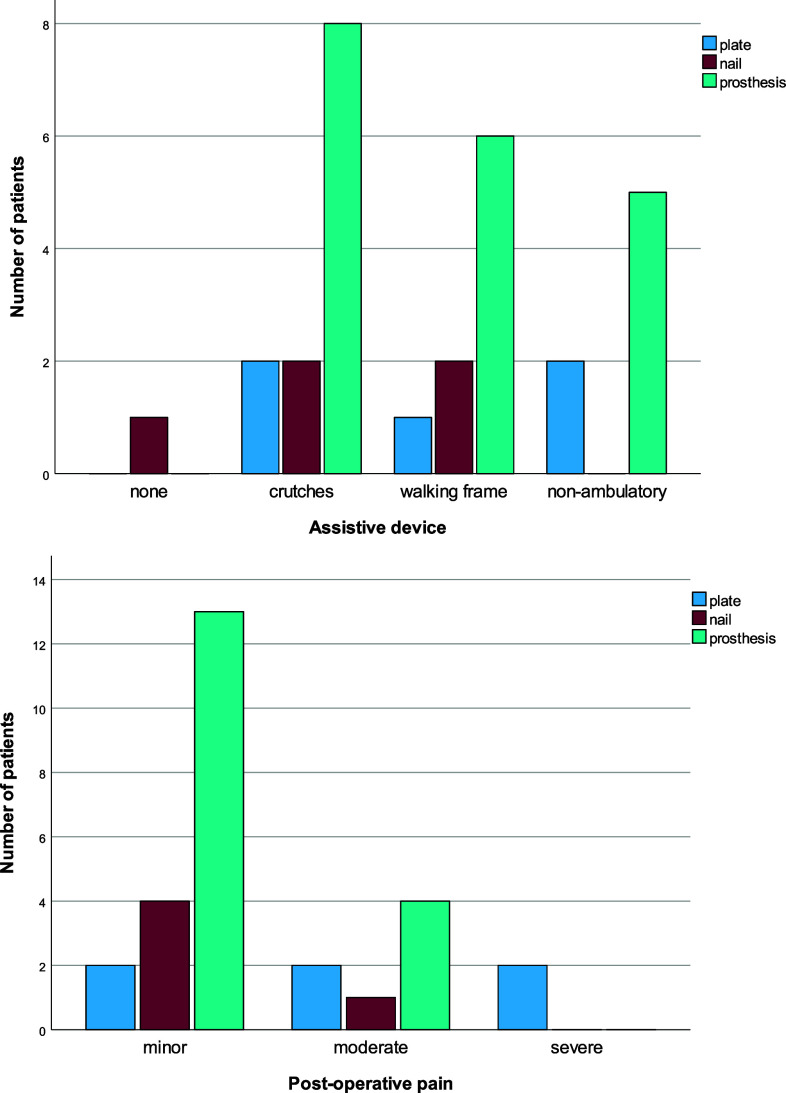
Functional outcome regarding observed ambulatory capacity (A) and reported pain (B), at an average of 6 weeks after surgery for distal femoral metastasis.

Surgical treatment resulted in restoration of the ambulatory capacity and diminished pain levels in most patients, with minor differences between treatment methods regarding pain levels. Data on postoperative ambulatory capacity and pain levels at 6 weeks were available for 29 patients. As shown in Figure 3a, ambulatory capacity was restored in 8/10 patients operated with OS and in 14/19 patients with EP (*p* = 0.395). At the same time, 2 of 11 patients in the OS group had severe pain (both operated with plate OS), but none in the EP group (*p* = 0.072, Figure 3b). Mean hospital stay was 7 days (range 2–12) in the EP group and 5 days (range 3–8) in the OS group.

In the EP group, who had a longer survival, the ambulatory capacity could also be retrieved for 10 patients at a longer follow-up interval of 3–9 months post-operatively, and it was retained for 7/10 patients, 5 of whom had independent ambulation without any assistive device. The pain levels were minor or absent for all 7 patients.

## Discussion

The goal with surgery for a pathological fracture is a stable fixation that can handle immediate weight-bearing and offer pain relief and restoration of the ambulatory capacity, at the expense of a reasonably low risk for complications. While there is a lot of evidence from previous studies regarding the outcome of surgery in the proximal and middle part of the femur, as well as the humerus, there is a notable paucity of data regarding the distal femur, due to the fact that it is a less common localization for metastatic lesions [4]. In the present study, we show that the type of surgical reconstruction is associated with different outcomes regarding the local complication rate and their management, with tumor relapses in plate OS and infections in EP, but generally comparable functional results.

Our study has shortcomings, being retrospective in nature, as are all other studies in this field. It thus suffers from selection bias regarding the choice of surgical intervention, lack of a standardized follow-up schedule, and missing data for some parameters. This accounts for the mainly descriptive nature of our analysis. At the same time, to our knowledge, it is the largest study reporting on the treatment of patients with such fractures, and where both OS methods and EP cases are included in adequate numbers to allow direct comparison. Some evidence can be derived from previously published bone metastases cohorts where patients with distal femoral lesions were included. In a study of 110 patients with femoral metastases, 13 of them were operated on with a prosthesis for distal femoral lesions, and 4/13 had a complication (3 infections leading to revision and 1 loosening that did not require treatment), whereas 16 had nails, and one required amputation due to tumor progression [2]. In another study of 192 patients operated for metastatic disease, 16 were located in the distal femur, and 10 of them were treated with plate OS, of which 2 were revised (one for tumor progression and one due to a stress fracture), whereas one had an EP and had no complication. There were also 5 patients treated with other methods, mainly cyrettage and cementation, which are outside the scope of the analysis [3]. The results of all three available studies are shown in Table 2.

**Table 2:** Overview of the studies reporting on local complications of patient cohorts operated for metastatic disease of the distal femur.

Study (year)	Surgery (number of fractures and type of reconstruction)	Local failures and complications
Wedin et al. (1999) [3]	11 (10 plate OS, 1 EP)	2 OS revisions (1 due to tumor progression, 1 due to stress fracture).
Mavrogenis et al. (2012) [2]	29 (16 nail OS, 13 EP)	1 OS (nail) amputation due to progression, 3 EP revisions due to infection.
		One EP aseptic loosening (no treatment).
Mellgren (present study)	48 (10 plate OS, 9 nail OS, 19 EP)	3 plate OS revisions (2 due to tumor progression, 1 due to technical error), 5 EP revisions (3 due to infection, 1 due to fracture, 1 due to technical error).
		3 infections (2 plate OS, 1 nail OS) treated with wound washout and/or antibiotics. One local recurrence (EP) treated with radiotherapy. Two peroneal nerve palsies (1 plate OS, 1 EP).

The main challenge for pathological fractures in this area is the poor soft-tissue coverage, which predisposes to infection. Furthermore, potential soft-tissue invasion by the tumor complicates the situation further. The local complication rate for EP in the distal femur is higher than in the proximal part [16]. Flap reconstruction may be needed in cases of extensive primary resection or secondary surgery for infection [17, 18]. Our results confirm this notion, where 3 of the 29 EP were revised due to infection, and one of them finally resulted in amputation. EP has the potential for better local tumor control, which was also evident in the present cohort, with no revision for local tumor recurrence despite the longer survival time of the patients in this group. This is justified since the excision of the tumor is much more radical than in cases of OS, where, at best, an intralesional resection is performed, sometimes even simple stabilization without any tumor removal.

The secondary challenge, encountered in cases of OS, is that the metaphyseal bone involved by the tumor can be of poor quality, and pain due to instability, tumor progression, or even secondary failure due to loss of fixation may be seen. Revision due to tumor progression was the case for 2 of the 8 plate OS in our series, and is comparable to a previous study where 2 of the 10 plate OS cases were revised for the same reason [3]. Furthermore, the poor bone quality contributed to the technical error of screw penetration of the joint, since tactile feedback and intraoperative imaging were difficult. The fact that plate OS was associated with a higher pain level at follow-up is probably explained by the inadequate biomechanical stability and residual tumor progression. Notably, we had no cases of nail failure, and in the previous report, only 1 of 16 nails failed [2]. Thus, in minor dislocations and preserved bone stock, nails appear as a safe choice, at the same time allowing for minimally invasive surgery.

Despite the presence of a high rate of complications, the overall surgical outcome was satisfactory, with restoration of the ambulatory capacity and pain reduction in most patients. This, admittedly limited, description of the functional outcome is also a novelty of this study. Some patients remained dependent on a wheelchair or bedridden after surgery, but this had to do with their poor general condition and limited ability for rehabilitation. Even in these cases, surgery offered good pain control apart from some patients with plate OS. No other significant difference in the functional outcome between the two major types of surgical reconstruction in this cohort. Patients in good condition and long survival have excellent functional outcomes when operated with an EP, with minor pain and very good ambulatory capacity, corroborating previous data [13, 14]. This indicates that the post-operative result is predictably good when the surgeon chooses the correct implant for the individual patient, and survival estimation tools may be used to facilitate this choice [12].

## Conclusion

Our results suggest that in patients with extensive skeletal defects or displaced pathological fractures due to metastatic lesions of the distal femur, EP should be considered when the oncological prognosis is good. In non-displaced fractures and minor defects, retrograde nailing may be the first option. Plate OS should generally be reserved for patients with poor survival, where the bone quality does not allow for nail OS. Given its significant limitations, we believe that this retrospective study provides valuable insights into surgical management and clinical outcomes of patients with metastatic lesions of the distal femur, and offers a basis for decision-making in the selection of surgical interventions that can contribute to better patient care.

## Data Availability

Anonymized data can be provided by the corresponding author upon reasonable request.
